# The Pattern Glare Test: Further Exploration of Methodological and Demographic Factors

**DOI:** 10.1167/iovs.66.14.49

**Published:** 2025-11-19

**Authors:** Lenka Jurkovičová, Julie Páleník, Alexandra Ružičková, Robert Roman, Milan Brázdil

**Affiliations:** 1 First Department of Neurology, St. Anne's University Hospital and Medical Faculty of Masaryk University, Brno, Czech Republic; 2 CEITEC – Central European Institute of Technology, Masaryk University, Brno, Czech Republic; 3 Department of Psychology, Faculty of Arts, Masaryk University, Brno, Czech Republic; 4 Laboratory for the Experimental Research of Religion, Faculty of Arts, Masaryk University, Brno, Czech Republic

**Keywords:** pattern sensitivity, perceptual distortions, visual discomfort, pattern glare, cortical hyperexcitability

## Abstract

**Purpose:**

The Pattern Glare Test (PGT) is a widely used tool for assessing visual discomfort and perceptual distortions elicited by repetitive, high-contrast patterns. It has been applied across diverse populations and research fields, including neuroscience, cognitive psychology, optometry, orthoptics, and ophthalmology. Despite its broad use, considerable variation exists in how the PGT is implemented, particularly regarding stimulus parameters, participant characteristics, and scoring methods. Such variability introduces potential confounding factors that may obscure the psychological and neurological mechanisms underlying pattern-induced visual discomfort.

**Methods:**

We analyzed PGT data from 184 neurotypical young adults (72 males and 112 female; mean age = 24.29 years, SD = 4.75) to examine methodological influences on reports of associated visual distortions (AVDs) and discomfort. Our main analyses utilized cumulative link mixed models and cluster analysis of intraindividual responses.

**Results:**

Significant effects of trial progression and sex (biological; male/female) on subjective responses have been revealed, with the effect of sex not accounted for by migraine occurrence. Substantial overlap among color-related AVDs supported their consolidation into a single category. Response patterns have been consistent across participants, emphasizing the robustness of the effect of spatial frequencies in the PGT.

**Conclusions:**

These findings demonstrate that the PGT is sensitive to individual differences in visual discomfort, while also highlighting the impact of several confounding variables that require careful consideration. This underscores the importance of methodological standardization and supports the need for future research to refine scoring strategies, thereby enhancing the test's reliability and interpretability in both clinical and research contexts.

The Pattern Glare Test (PGT) is a widely used tool in visual research, primarily utilized to evaluate susceptibility to visual discomfort.^1^^,^^2^ It was first developed by Arnold J. Wilkins and colleagues in the 1980s,^3^ with its standardized version—the Wilkins and Evans Pattern Glare Test—officially published in 2001.^4^ Many variants of the test, however, have been in use since.^5^^–^^16^

The PGT is grounded in the study of “pattern glare,” a phenomenon where viewing repetitive, high-contrast patterns, such as black-and-white gratings, induces visual discomfort and perceptual distortions.^17^ During the test, observers are presented with striped patterns of varying spatial frequencies, including the most aversive frequency of approximately 3 cycles per degree (cpd), to assess their susceptibility to visual discomfort and related effects. These effects often include the perception of colors, motion, or shapes that are not actually present in the stimulus and may even trigger symptoms such as headaches, eye strain, or nausea in some individuals.^1^

The pattern glare phenomenon, resulting in visual discomfort, is attributed by some authors to hyperexcitability in the visual cortex, where certain patterns excessively stimulate its neurons.^3^^,^^5^^,^^6^^,^^9^^,^^10^^,^^18^^–^^21^ The level of induced discomfort varies between individuals and is frequently elevated in conditions such as visual stress,^22^ migraine,^18^^,^^23^^–^^26^ epilepsy,^27^^–^^29^ stroke,^30^ or dyslexia.^31^^,^^32^ However, research in this area has also documented significant differences among healthy individuals.^9^^,^^10^^,^^14^^,^^33^^,^^34^

Despite its widespread application in neuroscience, cognitive psychology, and ophthalmology research,^13^^,^^20^^,^^21^^,^^35^^–^^37^ the methodologies using PGT demonstrate considerable variability across studies, reflecting diverse research objectives, theoretical frameworks, and experimental designs. Importantly, the influence of potential moderating factors, such as participant characteristics and stimulus properties, is accounted for to varying extent. This inconsistency may limit the ability to draw generalizable conclusions and to fully understand the psychological and neurological mechanisms underlying visual discomfort, and also poses challenges for cross-study comparisons.

The age distribution of participants across the PGT literature ranges from young students to different cohorts of productive-age individuals to seniors; some studies were even conducted on children. Because there is evidence that the effect of pattern glare decreases significantly with age,^2^ the conclusions based on data from various cohorts should be interpreted with caution.

Similarly, the variability extends to the consideration of sex and gender. In life sciences research, either biological sex, gender, or both should be accounted for due to their impact on biological, environmental, social, and psychological processes, which may interact with study hypotheses.^38^ This is particularly relevant in studies of processes associated with the human brain, which exhibits significant sexual dimorphism influenced by testosterone.^39^ It is essential to use the terms “sex” and “gender” appropriately and to distinguish them in sample characteristics. Sex is a biological variable defined by characteristics encoded in DNA, such as reproductive organs and other physiological and functional characteristics. In contrast, gender refers to the social, cultural, and psychological characteristics associated with human male and female subjects through social context.^40^ Although the key PGT normative study by Evans and Stevenson^2^ assessed gender differences in pattern glare and found no significant effects, this conclusion was based on a relatively small sample (34 male and 43 female subjects) with a wide age range (10–90 years), including children, which limits its generalizability. In contrast, our prior research suggests that biological sex may significantly influence PGT outcomes in certain populations^41^ and requires further exploration.

In regard to test parameters, although some studies have demonstrated the importance of specific stimulus characteristics, such as contrast and color,^3^^,^^42^^,^^43^ viewing distance,^44^ or presentation order,^2^ these variables are not always clearly reported and controlled for in PGT studies, posing challenges for reproducibility. Additionally, numerous other potential confounding factors related to the physical, temporal, or spatial characteristics of the stimuli remain to be further examined.

In addition to the stimuli themselves, the subjective response scoring systems introduce a critical source of variability across study designs. Various versions of visual distortion lists are used. Some authors use the original list of seven symptoms recommended in the PGT manual,^4^ that is, colors, bending of lines, blurring of lines, shimmering or flickering, fading, shadowy shapes, and others. Other authors have modified the list to suit specific research objectives, either by adding or removing visual or somatic symptoms, or by subdividing the “colors” category into specific colors.^10^^,^^20^^,^^37^^,^^45^^–^^47^ These differences in scoring approaches can lead to divergent outcomes, and therefore, examining the potential influence of such methodological variability is warranted.

The current study explores potential moderating factors by analyzing empirical data from 184 young neurotypical participants. We examine several key PGT variables and their effects on participants’ subjective reports of core outcomes: perceptual distortions and discomfort. Whereas some methodological and participant-related factors have been examined previously, this study applies advanced statistical modeling to a large sample (*n* = 184) to extend current understanding of their influence on Pattern Glare Test outcomes. This approach aims to identify confounding factors and evaluate their influence on PGT results, ultimately informing refinements to the test's application in both research and clinical settings, while advancing understanding of the pattern glare phenomenon.

## Methods

We present a re-analysis of PGT data previously collected in our laboratory, focusing on the influence of potential confounding variables recorded during the experiment and on the internal structure of subjective responses. To our knowledge, this is the first such analysis reported in the context of the PGT. The data originate from the experiment detailed in Jurkovičová et al.,^41^ where the complete methodology, including other utilized methods, is described. Only the aspects that are most relevant to the PGT are described here. Contrary to the original study, no participants were excluded based on their responses to the PGT or incomplete magnetic resonance imaging (MRI) data to ensure the largest and most representative sample.

Inclusion criteria required participants to be 18 to 40 years old, have normal or corrected-to-normal vision, have no psychiatric or neurological disorders (except for migraine without aura and common headaches), have no history of migraine with aura, no MRI contraindications, and provide signed informed consent. Participants were recruited via a database of volunteers for neuroscience research and advertisements in university/social media. The sample included 122 students, plus 10 PhD-level students (71.7% of the sample in total). The study was approved by the Research Ethics Committee of Masaryk University. The study was conducted in accordance with relevant guidelines and regulations following the principles of the Declaration of Helsinki.

### Pattern Glare Test

**Figure 1.**
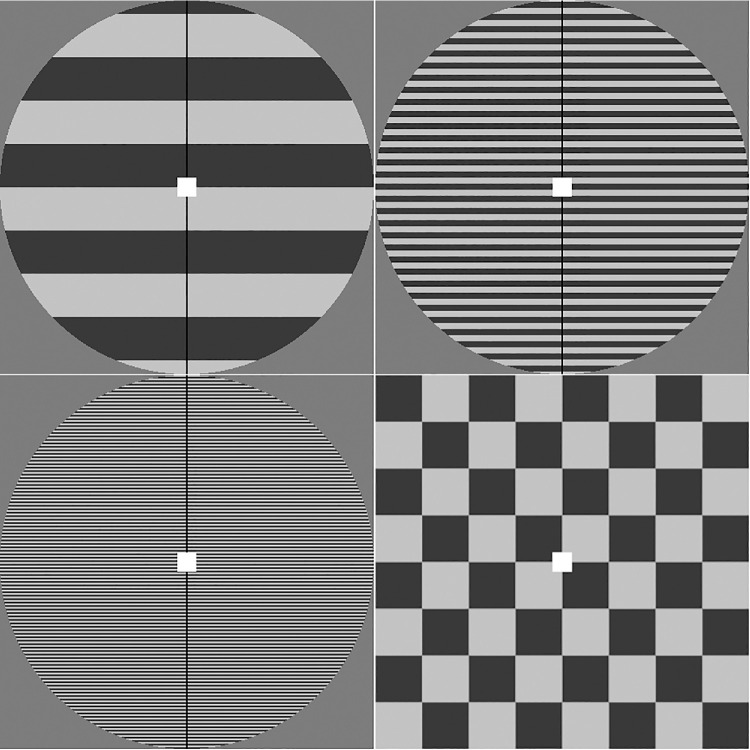
Stimuli used in the experiment. The *upper row* shows the 0.5 cycles per degree (cpd) and 3 cpd gratings. The *lower row* shows the 14 cpd grating and 0.5 cpd checkerboard, shown from *left* to *right*.

A computerized version of the PGT was administered in a shielded laboratory, following the methodology suggested by Braithwaite et al.^10^ Distance of 80 cm from the LCD screen was ensured using a chinrest. Light in the room was kept at a stable 35 lux. Four types of stimuli were presented: 3 achromatic square-wave gratings at different spatial frequencies (0.5 cpd, 3 cpd, and 14 cpd) and a black-and-white 0.5 cpd checkerboard in a square window, included to mitigate the possibility of persistent neural activity or afterimages influencing responses to subsequent stimuli (Fig. 1). Michelson contrast of the gratings was 0.7 as in the original test by Wilkins et al. in 1984.^3^

The stimuli were shown to participants in pseudorandomized order, where every stimulus was seen once during a block of four presentations and the same stimulus was never presented twice in a row (including at the beginning or end of a block of 4 stimuli). Each stimulus was presented 6 times, resulting in 24 trials overall.

Before each trial, a fixation dot was presented in the center of the screen for a random interval between 6 seconds and 8 seconds (interval length picked from a uniform distribution). After this, 1 of the 4 stimuli was presented for 12 seconds, with the fixation dot remaining on the screen. After answering the questions about their experience viewing the pattern (see below), a blank screen was shown for 8 seconds before the start of the next trial.

Three kinds of responses were collected from participants in each trial. First, the participants selected all associated visual distortions (AVDs) they experienced while viewing the stimulus from a list. These included ocular pain, shadowy shapes, shimmering, flickering, bending, blurring, nausea, and red, yellow, green, or blue color. A printed-out list of AVDs, which included their definitions, was provided to participants before the task and was available to them during the entire procedure.

Second, participants were asked to determine whether the AVDs were present more on one side of the screen, left or right, or on both sides roughly equally (lateralization). Last, participants rated their level of discomfort on a 11-point scale ranging from extreme discomfort (–5), through neutral experience (0) to extreme comfort (5).

### Data Analysis

All the data analyses were conducted in RStudio (version 2024.04.2+764) using R software version 4.4.1. The data and R script are publicly available at 10.5281/zenodo.15051368.

First, the internal structure of the PGT was investigated by analyzing the data on the level of single trials. The distribution of different AVDs on the four types of stimuli was compared using the chi-squared test. The tendency of pairs of AVDs to co-occur on a single trial was quantified using the overlap or Szymkiewicz–Simpson coefficient. The advantage of this metric over other similarity measures, such as the phi coefficient, is that it equals one when one of the phenomena is a subset of the other. Comparatively rare phenomena therefore can still have high degrees of overlap with more common ones.

Next, two PGT scores were computed in accordance with previous works: average number of endorsed AVDs (either counting the 4 reportable colors separately or as one AVD) and the average comfort rating, both averaged per trial of a stimulus type. These scores were compared using the Friedman test, a nonparametric equivalent of a repeated measures ANOVA, and post hoc analyses were conducted using paired Wilcoxon signed-rank tests with Bonferroni adjusted *P* values.

We also investigated whether individuals exhibited distinct response patterns across the four different stimuli. Average AVDs were converted into relative scores by dividing each subject's PGT score for a certain stimulus type by the sum of their PGT scores across all four stimuli. K-means cluster analysis was then conducted using Euclidean distances of these relative PGT scores. The number of clusters was established using a combination of the elbow method, average silhouette, and the gap statistic method (based on the first local maximum).

Finally, responses on single trials, both AVD sums and comfort scores, were predicted using cumulative link mixed models (CLMMs) with a logit link function from the “ordinal” R software package.^48^ CLMMs are an extension of logistic regression. Whereas logistic regression predicts binary outcomes, CLMMs estimate separate thresholds for each level of an ordinal response variable, thereby modeling the probability that a response falls within a given ordered category. This approach accounts for the ordinal nature of the data without assuming equal distances between categories, providing a more accurate representation of the observed responses. Coefficients of a CLMM represent log-odds of the ordinal response variable being in a category one higher per unit of the predictor. In this study, we report exponentiated coefficients, converting them into odds ratios (ORs).

The predictor variables included the presented stimulus, trial number, lateralization of reported distortions, biological sex, age, jitter of the stimulus onset, previous stimulus (i.e. stimulus presented in the preceding trial), presence of migraine, and two-way interactions of sex with migraine and age with migraine, along with a random effect of the subject. Due to the inclusion of the “previous stimulus” variable, the first trial of each subject was excluded from the analysis.

A major assumption of CLMMs are proportional odds, meaning that the effects of predictors are equivalent across all levels of the response variable. Because the “ordinal” package does not implement a formal test for the proportional odds assumption in the case of CLMMs, we investigated this assumption by fitting separate binomial generalized linear mixed models (GLMMs) for each level of the response variable and comparing the model coefficients.

## Results

Demographic information of the sample has been summarized in Table 1. The analyzed sample consisted of 184 subjects (self-reported biological sex = 72 male and 112 female subjects). The average age of subjects was 24.29 years (SD = 4.75). The age distribution was positively skewed (1.42), indicating a higher proportion of younger participants. Most respondents were native Czech (135) or Slovak (43) speakers. Other reported native languages included Ukrainian (1), Romanian (1), Belarussian (2), and Russian (1), whereas one participant claimed both Czech and Slovak as native languages. All participants were fluent in Czech. Participants reported the number of hours slept before the experiment (M = 6.91, SD = 1.30) and on their typical night (M = 7.45, SD = 0.85). Twenty-nine participants reported experiencing symptoms of migraine without aura at some point during the last 10 years, whereas 154 reported no symptoms. One subject did not respond to the migraine-related question.

### Internal Structure of the PGT

**Figure 2.**
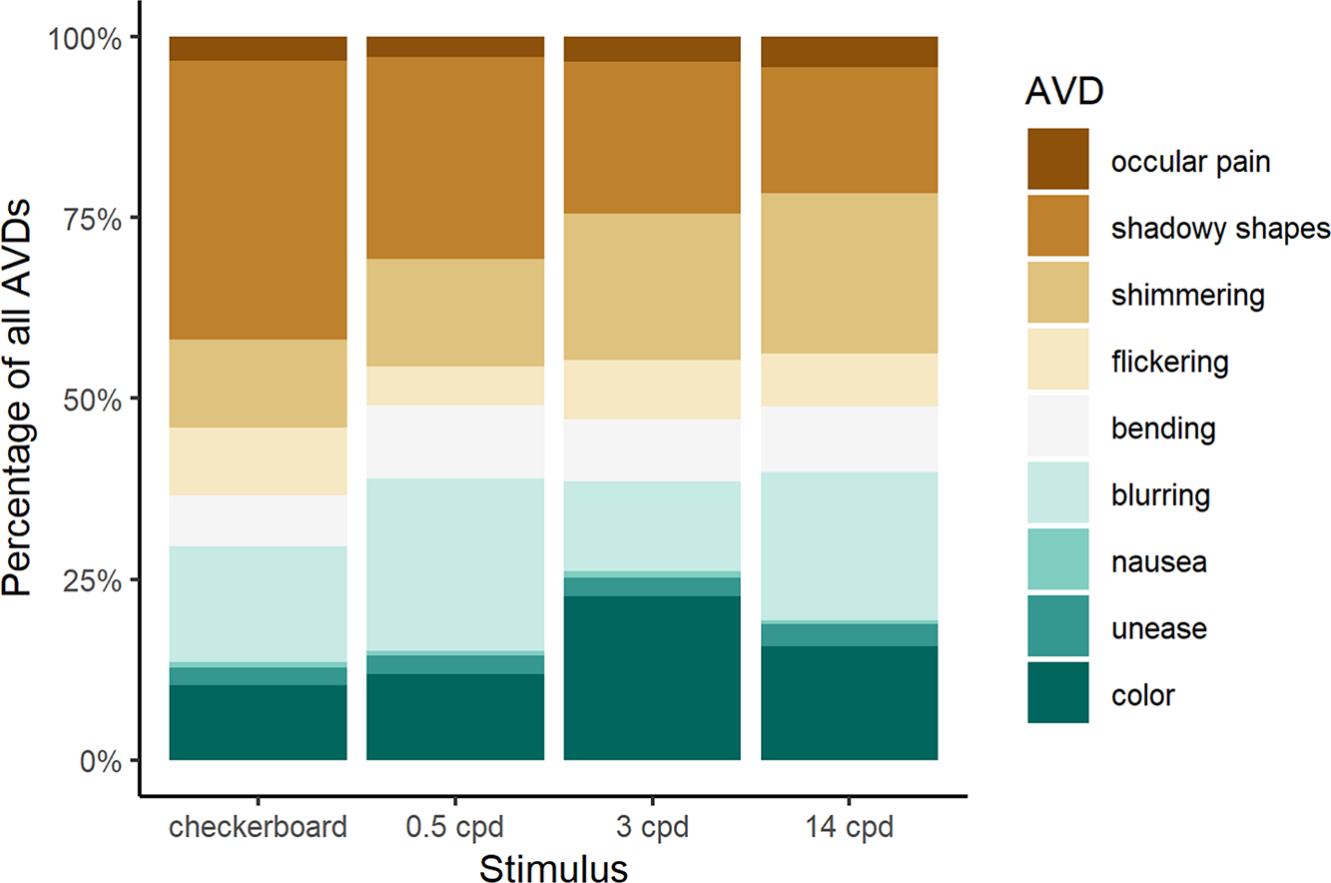
Distribution of AVDs on different stimuli.

At least one AVD was reported on 74.21% of all trials (52.86% of 0.5 cpd trials, 89.76% of 3 cpd trials, 93.02% of 14 cpd trials, and 61.12% of checkerboard trials). The specific type of AVD experienced differed significantly across the four stimuli (χ^2^ = 685.95, df = 33, *P* < 0.001). When the colors were treated as a single AVD category, the trend remained highly significant (χ^2^ = 383.41, df = 24, *P* < 0.001). Colors were experienced more frequently for 3 cpd gratings than for other stimuli, whereas shadowy shapes were most commonly observed in the control checkerboard stimulus (see Fig. 2).

**Figure 3.**
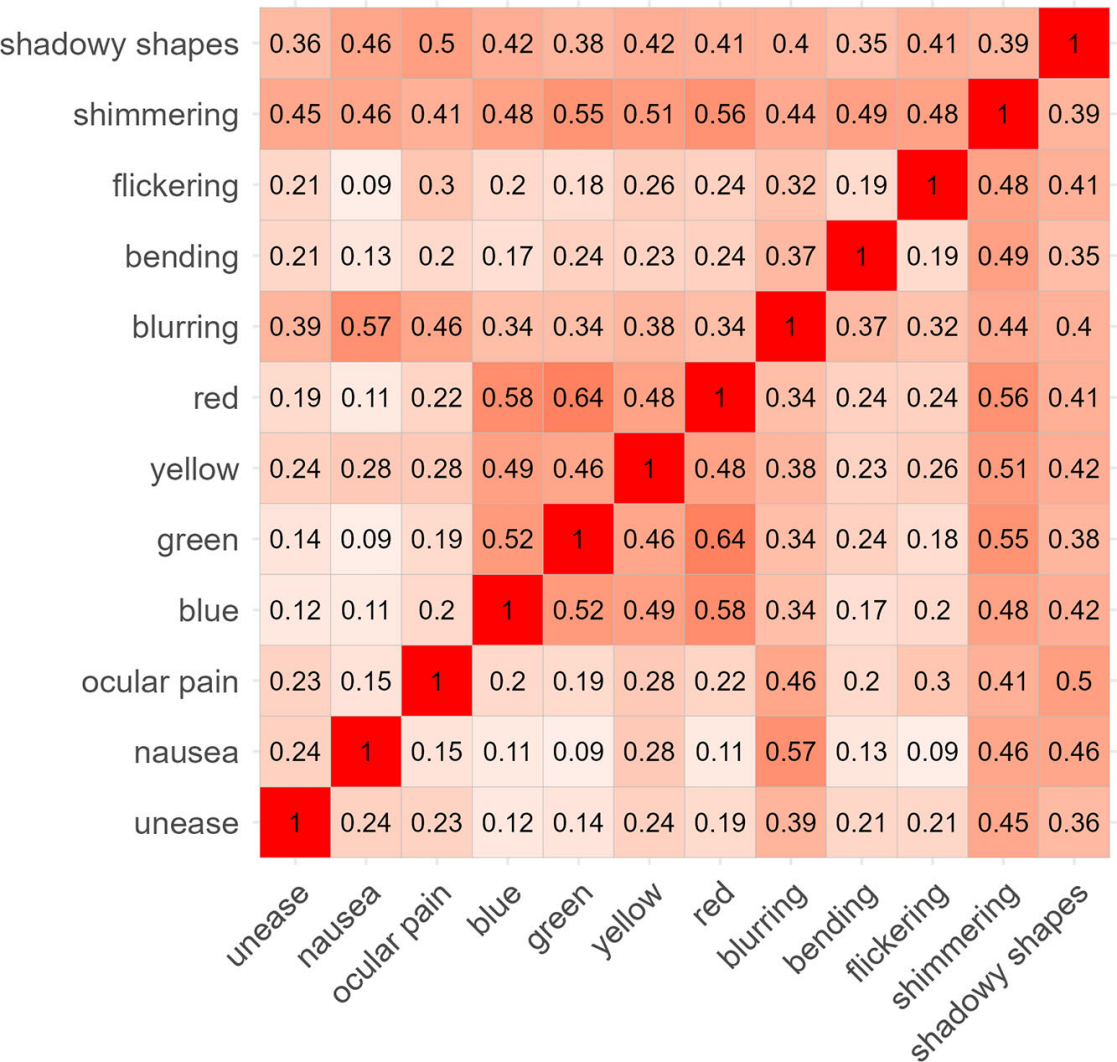
Overlap coefficient values for pairs of AVDs.

The matrix of overlap coefficients of different pairs of AVDs is presented in Figure 3. The color variables (red, yellow, green, and blue) display a high degree of overlap (all ≥ 0.46), which justifies the decision in some previous studies to combine them into a single “color” variable.

**Figure 4.**
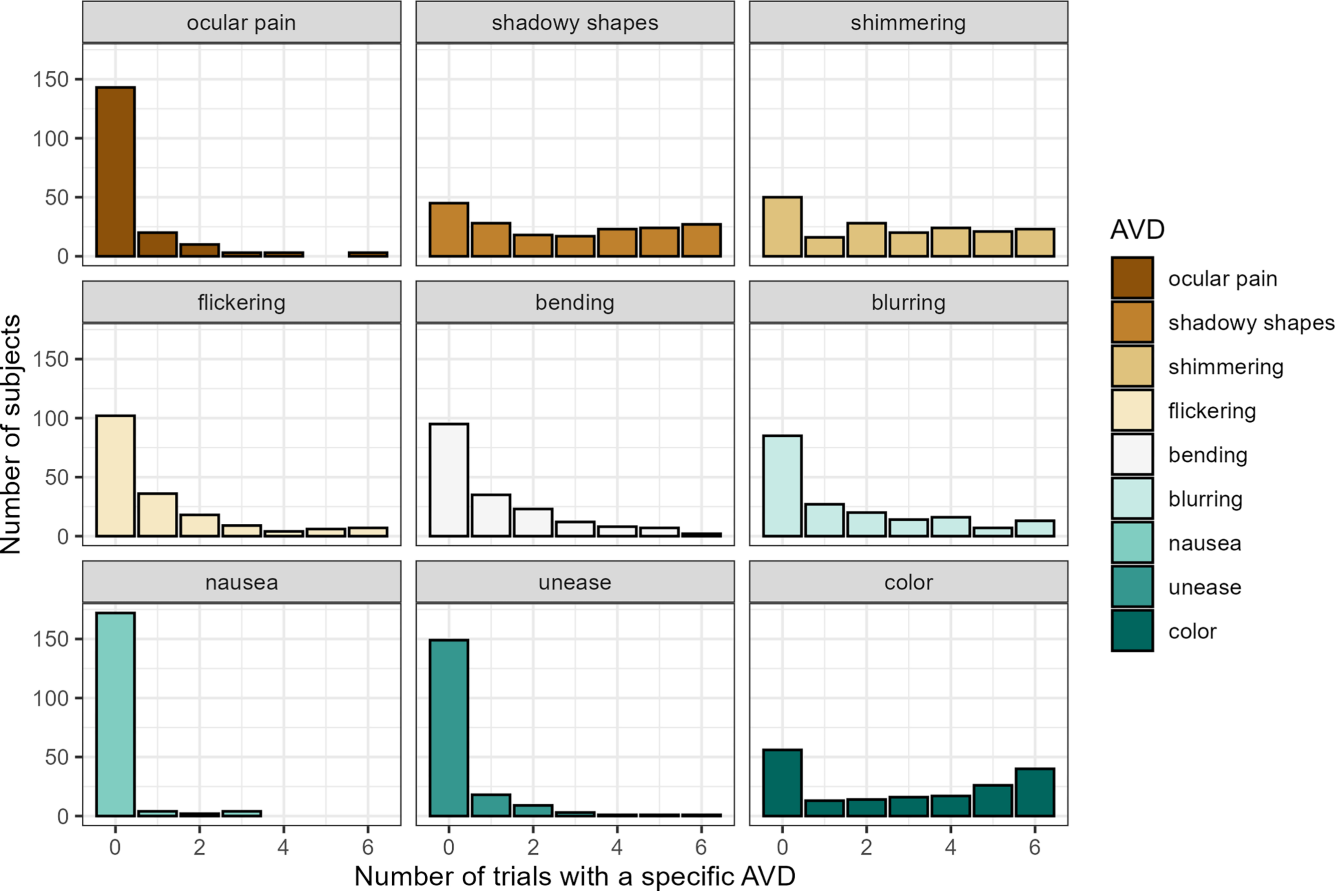
Histograms showing the proportion of target 3 cpd trials in which subjects experienced specific AVDs.

Figure 4 shows the distributions of specific AVDs on the 3 cpd grating (ranging from 0 = the subject never experienced this AVD on the 3 cpd; to 6 = the AVD was experienced on each of the 3 cpd presentations). All the distributions have a mode of zero, although shadowy shapes and colors (2 of the most common AVDs) also show a trend toward bimodality with modes at the extremes of the variable range.

Lateralization of the perceived phenomena, that is, the side where the distortions were more prominent, was reported in 12.08% of all trials (7.3% on the left and 4.78% on the right side of the visual field). For the target 3 cpd stimulus, 124 of the 184 subjects (67.39%) never reported any lateralization. Moreover, 80 subjects (43.48%) did not report lateralization on any trial across all four stimulus types during the entire task.

### Overall PGT Scores

**Figure 5.**
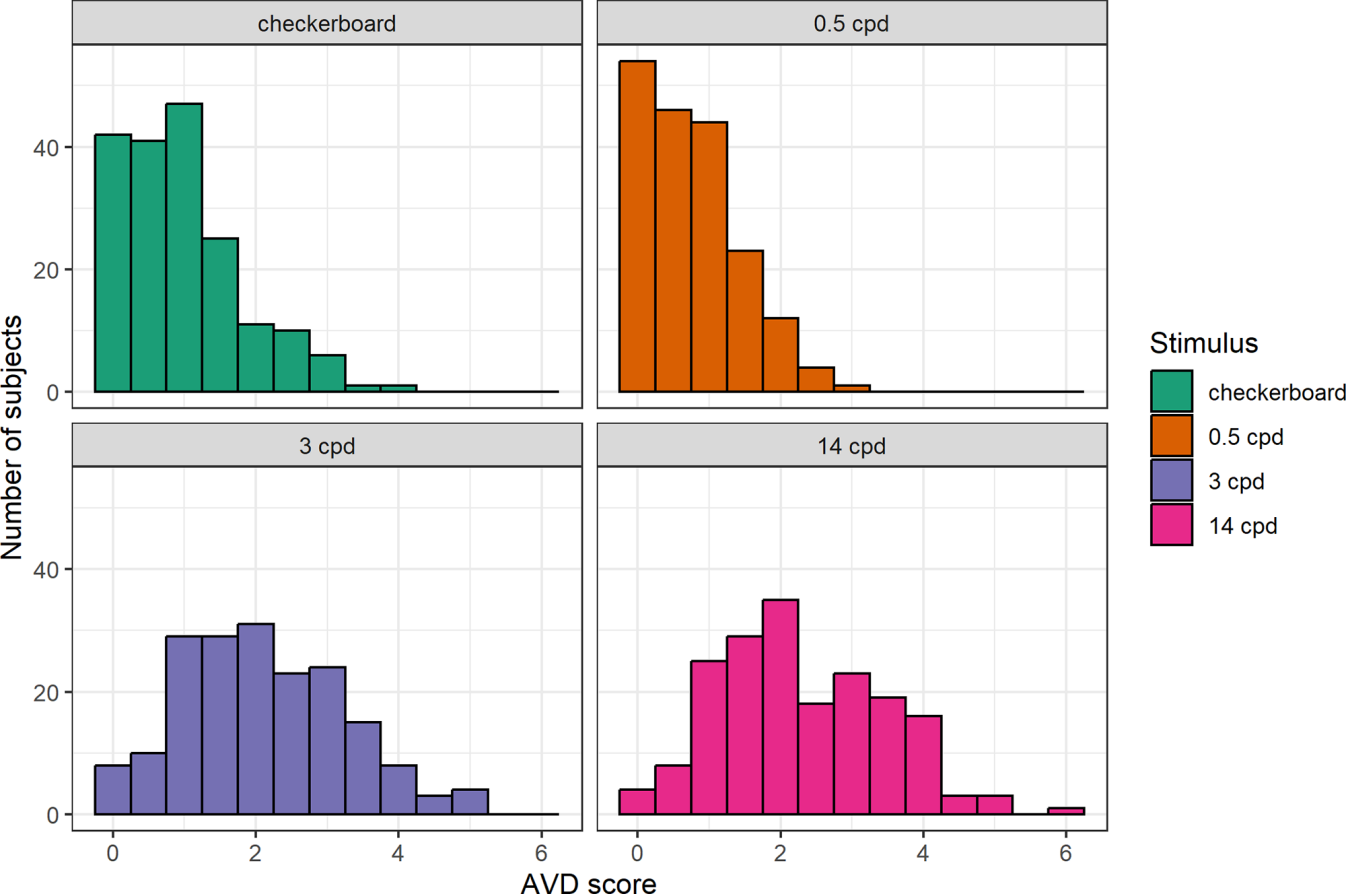
Distributions of overall AVD scores. The score represents the average number of endorsed AVDs per trial for each specific stimulus type.

Two overall PGT scores were computed: average number of AVDs and the average discomfort rating per trial for each of the four stimulus types. The distribution of overall AVD scores is shown in Figure 5. The scores for the 3 cpd and 14 cpd gratings were approximately normally distributed; however, the Shapiro-Wilk test was significant in both cases (*P* < 0.01), likely due to the large sample size.

The Friedman test was highly significant (χ^2^(3) = 373, *P* < 0.001). All paired Wilcoxon tests remained significant after correction (*P* < 0.01). The highest number of AVDs was reported on the 14 cpd grating (M = 2.82, SD = 1.16), followed by the 3 cpd grating (M = 2.11, SD = 1.15), the 0.5 cpd checkerboard stimulus (M = 0.95, SD = 0.83), and the lowest on the 0.5 cpd grating (M = 0.76, SD = 0.66).

**Figure 6.**
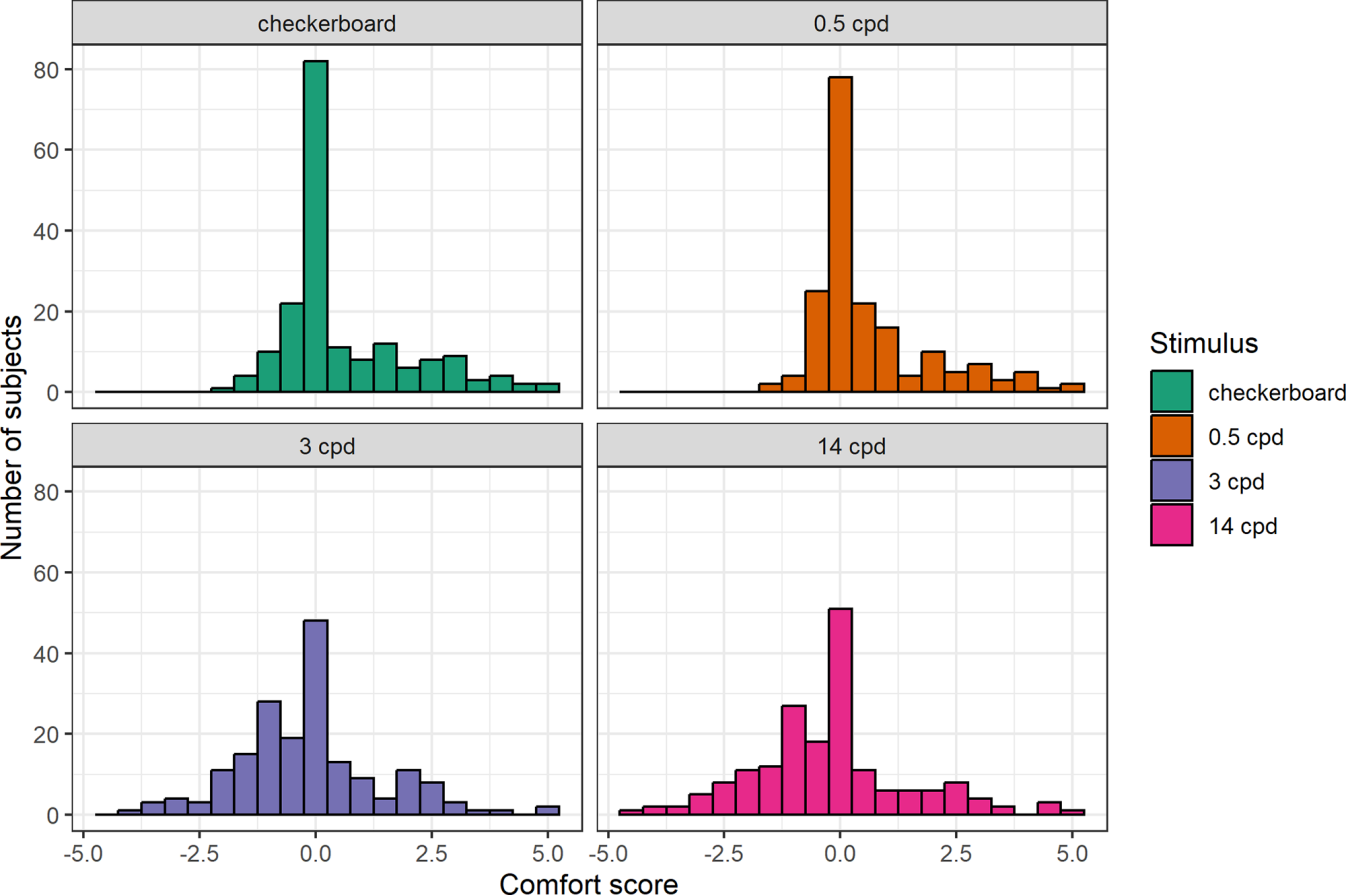
Distributions of average comfort scores per trial for each specific stimulus.

Average comfort ratings were computed analogously to the AVD scores. As illustrated by the distributions in Figure 6, a large portion of the sample found the control 0.5 cpd gratings and checkerboards neutral (with an average comfort rating of zero) or only slightly irritable. However, a subset of subjects rated these stimuli as comfortable (positive comfort ratings), indicating that some participants may have been using different responding criteria than others.

Comfort scores varied significantly across the four stimulus types, according to the Friedman test (χ^2^(3) = 129, *P* < 0.001). To mitigate the effect of responding criteria, we further analyzed the data using a chi-square test, recoding comfort scores as a binary variable: presence (comfort score ≥0) or absence (comfort score <0) of discomfort. The differences between stimuli remained highly significant (χ^2^(3) = 51.794, *P* < 0.001).

All post hoc comparisons (paired Wilcoxon tests) were significant at corrected *P* < 0.001, except for those between the 0.5 cpd grating and checkerboard, and between the 3 cpd and 14 cpd gratings. Lower discomfort was reported on checkerboard (M = 0.57, SD = 1.39) and 0.5 cpd gratings (M = 0.61, SD = 1.28) compared to the 3 cpd (M = −0.14, SD = 1.54) and 14 cpd gratings (M = −0.2, SD = 1.66).

Finally, to examine individual profiles of responding to the different PGT stimuli, AVD scores were converted into relative values, and a k-means cluster analysis was conducted based on their Euclidean distances. Analysis of the entire sample, using both the average silhouette and gap statistic methods, indicated the presence of two clusters. However, because one cluster included only six participants, these were deemed to be outliers and were excluded from the second iteration of the cluster analysis.

**Figure 7.**
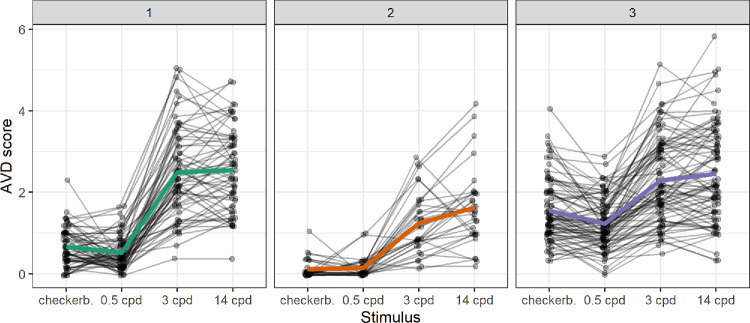
Individual profiles of the three clusters of PGT responses. Average cluster profiles are shown in *color* (without *dots*).

Among the remaining sample of 178 subjects, both the elbow and the gap statistic method suggested three clusters, whereas the average silhouette indicated two clusters. To facilitate interpretation, we opted to conduct the analysis with three clusters. The results of the k-means cluster analysis are shown in Figure 7. Cluster 1 comprised 64 subjects, cluster 2 included 31 subjects, and cluster 3 contained the remaining 83 subjects.

### Regression Modeling of Visual Distortions and Comfort Ratings

The number of endorsed AVDs and comfort ratings on single trials were modeled using CLMMs. Only exponentiated coefficients (ORs) are reported. A summary of the CLMM results can be found in Table 2, whereas the coefficients of separately fitted GLMMs are provided in Supplementary Materials.

**Table 1.** Summary of Demographic Characteristics of the Analyzed Sample

	*N*	Percentage
Biological sex		
Male	72	39.13
Female	112	60.87
Age, y		
18–20	29	15.76
21–23	83	45.11
24–26	31	16.85
27–29	14	7.61
30–32	11	5.98
33–35	8	4.35
36–38	5	2.72
39–40	3	1.63
Native language		
Czech	135	73.37
Slovak	43	23.37
Other	6	3.26
Migraine without aura		
Yes	29	15.76
No	154	83.7
Missing value	1	0.54

**Table 2.** Exponentiated Coefficients (Odds Ratios) of the CLMMs Predicting the Number of AVDs Per Trial or Comfort Ratings

	Number of Endorsed AVDs	Comfort Ratings
Trial	1.012**	0.979***
Lateralization (left)	1.002	1.249*
Lateralization (right)	2.028***	1.567**
Sex (male)	0.163***	0.84
Age, y	0.936***	0.969***
Stimulus onset jitter	1.047	0.99
Prev. stim. (0.5 cpd grating)	1.103	1.046
Prev. stim. (3 cpd grating)	0.826*	1.102
Prev. stim. (14 cpd grating)	0.934	1.069
Migraine	1.583***	0.889
Stimulus (0.5 cpd grating)	0.714***	1.041
Stimulus (3 cpd grating)	6.714***	0.433***
Stimulus (14 cpd grating)	8.76***	0.381***
Sex (male) * migraine	0.941	0.955
Sex (male) * age	1.049***	1.029*

Statistical significance marked as follows: *P < 0.05, **P < 0.01, and ***P < 0.001.

The effect of the presented stimulus was highly significant (*P* < 0.001) in all cases, except for the contrast between 0.5 cpd checkerboards and 0.5 cpd gratings (OR = 1.041, *P* > 0.05). Additionally, we observed a consistent effect of trial number, with later trials associated with an increase in AVDs (OR = 1.012, *P* < 0.01) and greater discomfort (OR = 0.979, *P* < 0.001).

Although a statistically significant effect of lateralization was found, further examination of the separate binomial models indicated that the assumption of proportional odds was likely violated for these coefficients. Additionally, the effect of previous stimulus on the number of AVDs was present mostly for the thresholds between zero and one, and one and two distortions. This pattern indicates that the effect is not consistent across all levels of the outcome variable. Consequently, we recommend not interpreting this coefficient as is, as it may not accurately reflect the effect across the full range of AVDs.

The main effects of biological sex and migraine without aura were significant only for AVDs. Male subjects reported fewer AVDs than female subjects (OR = 0.163, *P* < 0.001), and subjects with migraine reported more AVDs than those without (OR = 1.583, *P* < 0.001). Age also significantly predicted responses to the PGT, although the direction of the effects was not as expected. Higher age was associated with fewer reported AVDs (OR = 0.936, *P* < 0.001) but greater discomfort (OR = 0.969, *P* < 0.001). However, the effect of age was significantly different between men and women (AVDs: OR = 1.049, *P* < 0.001 and comfort: OR = 1.029, *P* < 0.05), being weaker in men.

## Discussion

Our study aimed to comprehensively investigate the factors influencing the PGT by re-analyzing empirical data and addressing specific research gaps using a controlled dataset. The presented analysis offers several advantages over previously reported research. We utilized a fairly large sample, considering that the data were collected in a laboratory setting, with mostly balanced representation of biological sex. Our version of the PGT was also highly controlled, maintaining constant lighting conditions and fixed viewing distance from the stimulus.

When analyzing overall PGT scores that are commonly used in research, we discovered statistically significant differences between almost all pairs of stimuli. Whereas comfort scores differed between the control and high-frequency stimuli, AVD scores were significantly different even between the two low spatial frequency stimuli (0.5 cpd gratings and checkerboard) and the two higher frequency stimuli (3 cpd and 14 cpd gratings). However, an examination of the comfort rating distributions revealed a potential area for improvement. Our findings suggest that the commonly used bipolar comfort scale with both positive and negative values^9^^,^^35^^,^^42^^,^^49^ may introduce bias in responses, as some participants do not utilize the full range of the scale, with only a subset utilizing positive values. For future research, we therefore recommend using a simplified discomfort scale, which goes from neutral ratings to different levels of experienced discomfort, provided that clear and thorough instructions are given.

Although participants’ AVD score patterns could be clustered, we did not identify any cluster with a radically different profile of AVD scores. Notably, all 3 identified clusters followed a similar pattern: low response scores on the control stimuli with 0.5 cpd and higher responses for the stimuli with higher spatial frequencies. This finding further supports the validity and practical usefulness of the PGT, when using the aversive grating with approximately 3 cpd as a target stimulus and low-frequency grating as the control stimulus.^2^^,^^32^

Age was found to be a significant factor influencing subjective ratings, and thus confirming the results of the study by Evans and Stevenson.^2^ Interestingly, the direction of age effects differed between the two predicted variables; higher age was associated with fewer reported AVDs but greater discomfort. This finding should be interpreted with caution, given our age-related inclusion criteria.

The significant main effect of biological sex aligns with our previous analyses on a subgroup of 160 participants from the same research.^41^ Importantly, the present re-analysis demonstrated that the interaction between sex and migraine was not significant, indicating that excluding individuals with migraines from the sample does not eliminate the influence of sex on AVDs. At least in this sample of young participants from the general population, the effect of sex on AVDs persists even when migraine is accounted for, suggesting that other factors, such as hormonal influences or inherent differences in visual processing, may contribute to these effects. In future research, it would be useful to explore these mechanisms in more detail, possibly by examining hormonal fluctuations, sex differences in sensory processing, or genetic predispositions. Additionally, studies including a more diverse age range and clinical populations could help clarify whether these effects extend beyond the current sample.

An intriguing question raised by this study is the inconsistent effect of stimulus order. Presenting a stimulus immediately after the target 3 cpd grating resulted in fewer AVDs being reported both for the high-frequency 14 cpd grating and the control stimuli, but only in cases when the overall number of endorsed AVDs was already low. This finding contrasts with the previously reported effect of stimulus order by Evans and Stevenson,^2^ who compared two age-matched groups (*N* = 30 and 27, age = 60 ± 6 and 7 years, respectively) and found that presenting a 12-cpd grating immediately after the 3-cpd grating led to an increase in AVDs on the subsequent stimulus. A significant effect of presentation order was also reported in the study by Wilkins et al.^3^; however, it did not influence their primary hypothesis and was not investigated further. In contrast, Monger et al.^44^ (*N* = 100, mean age = 21.4 ± 2.3 years) reported no effect of stimulus order, although specific group details were not provided. Although the use of high-frequency stimuli is no longer recommended,^32^ due to their strong dependence on viewing distance, our regression analysis of data from 184 subjects suggests that these stimuli may have some influence on lower thresholds of AVD reporting. However, this effect was not consistent across all response levels and the assumption of proportional odds has been violated, so we advise caution when interpreting this finding. Nevertheless, it points to the usefulness of controlling for stimulus order in experimental designs. It might be beneficial to further investigate this effect by examining whether the inclusion of neutral stimuli between irritable gratings or the introduction of longer time intervals between stimuli presentations improves the consistency of behavioral responses.

Regression modeling of single trials further revealed additional effects of intervening variables on the PGT. The effect of trial number indicates that the phenomena associated with pattern glare, both AVDs and discomfort, become more pronounced as the task progresses. Although the per-trial effect is small—each additional trial increases the odds of a higher AVD score by a factor of 1.012—this effect accumulates over multiple trials. Over the course of 24 trials, this results in an approximately 33% increase in the odds of reporting an additional visual distortion. Thus, although the effect size per trial is modest, it becomes statistically and perceptually relevant in longer experimental paradigms involving irritable gratings. Given the length of the present task, it was sufficient to capture this cumulative effect. The progressive increase in reported AVDs and visual discomfort across repeated trials suggests that prolonged stimulation may modulate cortical responsiveness over time. One plausible explanation is that repeated stimulation induces cortical hyperexcitability, whereby sustained activation of visual cortical networks may increase excitatory and reduce inhibitory neural activity. Such progressive alterations in cortical excitability balance could account for the heightened discomfort observed with repeated exposure to aversive visual stimuli. This mechanism may underlie the exacerbation of visual stress symptoms during prolonged near-vision activities, such as reading or digital screen use.^50^^,^^51^ Nonetheless, alternative factors may contribute. The trend could reflect visual or attentional fatigue, which may lower perceptual thresholds for discomfort, or response bias induced by repeated symptom reporting, potentially increasing suggestibility or expectancy effects. Future studies could address these processes by varying inter-trial intervals or including neutral stimuli already suggested above, or adjusting the frequency of subjective ratings to distinguish neural from cognitive factors. Neurophysiological measures (e.g., electroencephalogram [EEG] or function MRI [fMRI]) would further examine the neural mechanisms by directly assessing cortical excitability changes.

Our results suggest that illusory colors experienced during the PGT often overlap within single trials. This variability is likely attributable to the individual differences in subjective responding criteria, for example, some participants may report only the most prominent illusory color, whereas others may select all colors they perceive. To enhance reliability, we support using them as a combined single phenomenon, as it was recommended in the original PGT manual by Wilkins and Evans.^4^ This decision is supported by the high degree of overlap observed among individual color AVDs (all ≥0.46).

The relative distribution of experienced AVDs varied significantly across the four types of stimuli used in this study, suggesting that different mechanisms may underlie their generation. Some AVDs may be more prone to confabulation by highly suggestible participants, whereas others could be more directly linked to the stimulus processing. To reduce the influence of responses caused by suggestibility, methods such as using low-spatial frequency pattern as a screening tool for a response bias in suggestible individuals are sometimes used.^2^^,^^37^ Nevertheless, the current findings highlight the need for a more systematic investigation of AVD lists to better distinguish perceptually driven effects from those influenced by individual response tendencies.

An additional observation was the occurrence of lateralized distortions in a minority of trials (12.08%). However, further model inspection suggested that the proportional odds assumption may not hold for these coefficients, and therefore we recommend interpreting these effects with caution.

### Limitations

Although we consider our results to be significant for the future development of the PGT, there are several factors that limit the generalizability of our study. Most notably, our sample consisted of subjects from a specific age range (18–40 years), with the age distribution in this range being skewed toward younger subjects. Additionally, a majority of the participants (71.7%) were university students, increasing the odds that the sample is not representative of the general population even in the given age range.

Next, we used a computerized version of the PGT, whereas the test is often presented in paper form.^19^^,^^20^^,^^33^^,^^44^ The mode of presentation may affect the test outcomes. Our analysis was naturally limited to the methodological factors that varied within the experimental design, while other parameters remained constant. Consequently, we could not examine the effects of factors such as presentation time, contrast, phrasing of questions, or interstimulus intervals.

Finally, there are limitations inherent to the methods of data analysis that were used. The separate binomial models indicated the violation of proportional odds assumption for some coefficients (i.e. lateralization and order effects), as we pointed out in the previous section. Further, our cluster analysis identified only a small group of outliers in its first iteration, before being recalculated on a sample with these subjects removed. This indicates that most of the variance between subjects in their responding patterns was represented by this outlying group, not by the clusters presented in the second iteration, after removing the outliers. These specific results should therefore be interpreted only cautiously.

## Supplementary Material

Supplement 1

## References

[1] Wilkins AJ. Visual Stress. In: *Oxford Psychology Series*. Oxford, England, UK: Oxford University Press; 1995. Available at: 10.1093/acprof:oso/9780198521747.001.0001.

[2] Evans BJW, Stevenson SJ. The Pattern Glare Test: a review and determination of normative values. *Ophthalmic Physiol Opt*. 2008; 28(4): 295–309. 18565084 10.1111/j.1475-1313.2008.00578.x

[3] Wilkins AJ, Nimmo-Smith I, Tait A, et al. A neurological basis for visual discomfort. *Brain*. 1984; 107(4): 989–1017. 6509314 10.1093/brain/107.4.989

[4] Wilkins AJ, Evans BJW. Pattern Glare Test Instructions. i.O.O. Sales Ltd; 2001. Available at: https://www.yumpu.com/en/document/view/25026312/pattern-glare-test-instructions.

[5] Huang J, Cooper TG, Satana B, Kaufman DI, Cao Y. Visual distortion provoked by a stimulus in migraine associated with hyperneuronal activity. *Headache*. 2003; 43(6): 664–671. 12786927 10.1046/j.1526-4610.2003.03110.x

[6] Huang J, Zong X, Wilkins A, Jenkins B, Bozoki A, Cao Y. fMRI evidence that precision ophthalmic tints reduce cortical hyperactivation in migraine. Cephalalgia; 2011; 31(8): 925–936. 21622479 10.1177/0333102411409076 PMC3132147

[7] Adjamian P, Holliday IE, Barnes GR, Hillebrand A, Hadjipapas A, Singh KD. Induced visual illusions and gamma oscillations in human primary visual cortex. *Eur J Neurosci*. 2004; 20(2): 587–592. 15233769 10.1111/j.1460-9568.2004.03495.x

[8] Allen PM, Hussain A, Usherwood C, Wilkins AJ. Pattern-related visual stress, chromaticity, and accommodation. *Invest Opthalmol Vis Sci*. 2010; 51(12): 6843. 10.1167/iovs.09-5086 20610842

[9] Braithwaite JJ, Broglia E, Brincat O, Stapley L, Wilkins AJ, Takahashi C. Signs of increased cortical hyperexcitability selectively associated with spontaneous anomalous bodily experiences in a nonclinical population. *Cogn Neuropsychiatry*. 2013; 18(6): 549–573. 23441857 10.1080/13546805.2013.768176

[10] Braithwaite JJ, Mevorach C, Takahashi C. Stimulating the aberrant brain: Evidence for increased cortical hyperexcitability from a transcranial direct current stimulation (tDCS) study of individuals predisposed to anomalous perceptions. *Cortex*. 2015; 69: 1–13. 25967083 10.1016/j.cortex.2015.03.023

[11] Perenboom MJL, Zamanipoor Najafabadi AH, Zielman R, Carpay JA, Ferrari MD. Quantifying visual allodynia across migraine subtypes: the Leiden Visual Sensitivity Scale. *Pain*. 2018; 159(11): 2375–2382. 30015708 10.1097/j.pain.0000000000001343 PMC6203424

[12] Qi X, Fan H, Yang X, et al. High level of pattern glare in major depressive disorder. *BMC Psychiatry*. 2019; 19(1): 415. 31864335 10.1186/s12888-019-2399-6 PMC6925875

[13] Dance CJ, Ward J, Simner J. What is the link between mental imagery and sensory sensitivity? Insights from aphantasia. *Perception*. 2021; 50(9): 757–782. 34463590 10.1177/03010066211042186 PMC8438787

[14] Haigh SM, Haugland AM, Mendoza LR, Montero M. Auditory discomfort in visually sensitive individuals. *Front Psychol*. 2023; 14: 1126481. 38098527 10.3389/fpsyg.2023.1126481 PMC10720311

[15] Laycox CA, Thompson R, Haggerty JA, Wilkins AJ, Haigh SM. Flicker and reading speed: effects on individuals with visual sensitivity. *Perception*. 2024; 53(8): 512–528. 38711325 10.1177/03010066241252066 PMC12952847

[16] Veitch JA, Miller NJ. Effects of temporal light modulation on individuals sensitive to pattern glare. *LEUKOS*. 2024; 20(3): 310–346.

[17] Conlon E, Lovegrove W, Barker S, Chekaluk E. Visual discomfort: the influence of spatial frequency. *Perception*. 2001; 30(5): 571–581. 11430242 10.1068/p2954

[18] Fong CY, Law WHC, Braithwaite J, Mazaheri A. Differences in early and late pattern-onset visual-evoked potentials between self- reported migraineurs and controls. *NeuroImage Clin*. 2020; 25: 102122. 31931401 10.1016/j.nicl.2019.102122 PMC6957816

[19] Wang M, Qi X, Yang X, et al. The pattern glare and visual memory are disrupted in patients with major depressive disorder. *BMC Psychiatry*. 2022; 22(1): 518. 35918667 10.1186/s12888-022-04167-9 PMC9344705

[20] Hui CLM, Wong SMY, Yu TYT, et al. Visual-stress-related cortical excitability as a prospective marker for symptoms of depression and anxiety in young people. *Eur Arch Psychiatry Clin Neurosci*. 2023; 273(5): 1051–1060. 35972556 10.1007/s00406-022-01469-7

[21] Torrens WA, Pablo JN, Shires J, Haigh SM, Berryhill ME. People with high schizotypy experience more illusions in the Pattern Glare Test: Consistent with the hyperexcitability hypothesis. *Eur J Neurosci*. 2023; 57(2): 388–399. doi:10.1111/ejn.15886. 36484768 PMC9847329

[22] Wilkins AJ, Evans BJW. Visual stress, its treatment with spectral filters, and its relationship to visually induced motion sickness. *Appl Ergon*. 2010; 41(4): 509–515. 19286164 10.1016/j.apergo.2009.01.011

[23] Marcus DA, Soso MJ. Migraine and stripe-induced visual discomfort. *Arch Neurol*. 1989; 46(10): 1129–1132. 2803073 10.1001/archneur.1989.00520460125024

[24] Wilkins AJ, Huang J, Cao Y. Visual stress theory and its application to reading and reading tests. *J Res Read*. 2004; 27(2): 152–162.

[25] Harle DE, Shepherd AJ, Evans BJW. Visual stimuli are common triggers of migraine and are associated with pattern glare. *Headache J Head Face Pain*. 2006; 46(9): 1431–1440. 10.1111/j.1526-4610.2006.00585.x 17040340

[26] Fong CY, Law WHC, Fahrenfort JJ, Braithwaite JJ, Mazaheri A. Attenuated alpha oscillation and hyperresponsiveness reveals impaired perceptual learning in migraineurs. *J Headache Pain*. 2022; 23(1): 44. 35382735 10.1186/s10194-022-01410-2 PMC8981672

[27] Wilkins AJ, Darby CE, Binnie CD. Neurophysiological aspects of pattern-sensitive epilepsy. *Brain*. 1979; 102(1): 1–25. 106922 10.1093/brain/102.1.1

[28] Wilkins AJ, Binnie CD, Darby CE. Visually-induced seizures. *Prog Neurobiol*. 1980; 15(2): 85–117. 6779350 10.1016/0301-0082(80)90004-0

[29] Radhakrishnan K, St. Louis EK, Johnson JA, McClelland RL, Westmoreland BF, Klass DW. Pattern-sensitive epilepsy: electroclinical characteristics, natural history, and delineation of the epileptic syndrome. *Epilepsia*. 2005; 46(1): 48–58. 10.1111/j.0013-9580.2005.26604.x 15660768

[30] Beasley IG, Davies LN. Susceptibility to pattern glare following stroke. *J Neurol*. 2012; 259(9): 1832–1839. 22289968 10.1007/s00415-012-6418-5 PMC3432783

[31] Evans BJW, Cook A, Richards IL, Drasdo N. Effect of pattern glare and colored overlays on a simulated-reading task in dyslexics and normal readers. *Optom Vis Sci*. 1994; 71(10): 619–628. 7877805 10.1097/00006324-199410000-00004

[32] Wilkins AJ, Allen PM, Monger LJ, Gilchrist JM. Visual stress and dyslexia for the practising optometrist. *Optom Pract*. 2016; 17: 103–112.

[33] Allen PM, Dedi S, Kumar D, Patel T, Aloo M, Wilkins AJ. Accommodation, pattern glare, and coloured overlays. *Perception*. 2012; 41(12): 1458–1467. 23586285 10.1068/p7390

[34] Gilchrist JM, Allen PM. Lexical decisions in adults with low and high susceptibility to pattern-related visual stress: a preliminary investigation. *Front Psychol*. 2015; 6: 449. 25926810 10.3389/fpsyg.2015.00449 PMC4396132

[35] Ward J, Hoadley C, Hughes JEA, et al. Atypical sensory sensitivity as a shared feature between synaesthesia and autism. *Sci Rep*. 2017; 7(1): 41155. 28266503 10.1038/srep41155 PMC5339734

[36] Ward J, Baykova R, Dyson B, et al. A distinct electrophysiological signature for synaesthesia that is independent of individual differences in sensory sensitivity. *Cortex*. 2021; 139: 249–266. 33894542 10.1016/j.cortex.2021.02.031

[37] Fong CY, Takahashi C, Braithwaite JJ. Evidence for distinct clusters of diverse anomalous experiences and their selective association with signs of elevated cortical hyperexcitability. *Conscious Cogn*. 2019; 71: 1–17. 30904823 10.1016/j.concog.2019.03.003

[38] Holdcroft A. Integrating the dimensions of sex and gender into basic life sciences research: methodologic and ethical issues. *Gend Med*. 2007; 4: S64–S74. 18156104 10.1016/s1550-8579(07)80048-9

[39] Jazin E, Cahill L. Sex differences in molecular neuroscience: from fruit flies to humans. *Nat Rev Neurosci*. 2010; 11(1): 9–17. 20019686 10.1038/nrn2754

[40] National Institutes of Health. Consideration of sex as a biological variable in NIH-funded research (NOT-OD-15-102). Published online 2015. Available at: https://grants.nih.gov/grants/guide/notice-files/NOT-OD-15-102.html.

[41] Jurkovičová L, Páleník J, Kudlička P, et al. Subjective visual sensitivity in neurotypical adults: insights from a magnetic resonance spectroscopy study. *Front Neurosci*. 2024; 18: 1417996. 39391756 10.3389/fnins.2024.1417996 PMC11465554

[42] Braithwaite JJ, Broglia E, Bagshaw AP, Wilkins AJ. Evidence for elevated cortical hyperexcitability and its association with out-of-body experiences in the non-clinical population: new findings from a pattern-glare task. *Cortex*. 2013; 49(3): 793–805. 22209090 10.1016/j.cortex.2011.11.013

[43] Monger LJ, Wilkins AJ, Allen PM. Pattern glare: the effects of contrast and color. *Front Psychol*. 2015; 6: 1651. 26579034 10.3389/fpsyg.2015.01651 PMC4621622

[44] Monger LJ, Shah D, Wilkins AJ, Allen PM. The effect of viewing distance on responses to the pattern glare test. *Clin Exp Optom*. 2016; 99(1): 47–50. 26875852 10.1111/cxo.12364

[45] Allen PM, Hussain A, Usherwood C, Wilkins AJ. Pattern-related visual stress, chromaticity, and accommodation. *Invest Ophthalmol Vis Sci*. 2010; 51(12): 6843–6849. 20610842 10.1167/iovs.09-5086

[46] Hollis J, Allen PM. Screening for Meares–Irlen sensitivity in adults: can assessment methods predict changes in reading speed? *Ophthalmic Physiol Opt*. 2006; 26(6): 566–571. 17040420 10.1111/j.1475-1313.2006.00401.x

[47] Shepherd AJ, Hine TJ, Beaumont HM. Color and spatial frequency are related to visual pattern sensitivity in migraine. *Headache*. 2013; 53(7): 1087–1103. 23464876 10.1111/head.12062

[48] Christensen RHB. Ordinal: regression models for ordinal data. Published online August 19, 2024. Available at: https://cran.r-project.org/package=ordinal.

[49] Ten Brink AF, Bultitude JH. Visual sensitivity in complex regional pain syndrome and fibromyalgia: an online study. *Perception*. 2022; 51(3): 187–209. 35236184 10.1177/03010066211072641 PMC8958570

[50] Wilkins AJ, Evans BJW. *Vision, Reading Difficulties, and Visual Stress*. New York, NY: Springer International Publishing; 2022.

[51] Tyrrell R, Holland K, Dennis D, Wilkins A. Coloured overlays, visual discomfort, visual search and classroom reading. *J Res Read*. 1995; 18(1): 10–23.

